# *In silico* Docking Analysis for Blocking JUNO‐IZUMO1 Interaction Identifies Two Small Molecules that Block *in vitro* Fertilization

**DOI:** 10.3389/fcell.2022.824629

**Published:** 2022-04-05

**Authors:** Nataliia Stepanenko, Omri Wolk, Enrica Bianchi, Gavin James Wright, Natali Schachter-Safrai, Kiril Makedonski, Alberto Ouro, Assaf Ben-Meir, Yosef Buganim, Amiram Goldblum

**Affiliations:** ^1^ Department of Developmental Biology and Cancer Research, Faculty of Medicine, The Institute for Medical Research Israel-Canada, Hebrew University of Jerusalem, Jerusalem, Israel; ^2^ Laboratory of Molecular Modeling and Drug Discovery, Faculty of Medicine, School of Pharmacy, The Institute for Drug Research, Hebrew University of Jerusalem, Jerusalem, Israel; ^3^ Department of Biology, Hull York Medical School, York Biomedical Research Institute, University of York, York, United Kingdom; ^4^ Infertility and IVF Unit, Department of Obstetrics and Gynecology, Hadassah Ein-Kerem Medical Center and Faculty of Medicine, Hebrew University of Jerusalem, Jerusalem, Israel

**Keywords:** non-hormonal contraceptives, docking, *in vitro* fertilization, JUNO–IZUMO1 interaction, human sperm penetration assay

## Abstract

Combined hormone drugs are the basis for orally administered contraception. However, they are associated with severe side effects that are even more impactful for women in developing countries, where resources are limited. The risk of side effects may be reduced by non-hormonal small molecules which specifically target proteins involved in fertilization. In this study, we present a virtual docking experiment directed to discover molecules that target the crucial fertilization interactions of JUNO (oocyte) and IZUMO1 (sperm). We docked 913,000 molecules to two crystal structures of JUNO and ranked them on the basis of energy-related criteria. Of the 32 tested candidates, two molecules (i.e., Z786028994 and Z1290281203) demonstrated fertilization inhibitory effect in both an *in vitro* fertilization (IVF) assay in mice and an *in vitro* penetration of human sperm into hamster oocytes. Despite this clear effect on fertilization, these two molecules did not show JUNO–IZUMO1 interaction blocking activity as assessed by AVidity-based EXtracellular Interaction Screening (AVEXIS). Therefore, further research is required to determine the mechanism of action of these two fertilization inhibitors.

## Introduction

During the 20th century, prevention of unwanted pregnancies became a major concern for both individual women and society as a whole, resulting in the development of the first hormonal contraceptive that went into market in 1960. Since then, all orally administered contraceptives are composed of combinations of steroid hormones from the progestogen and estrogen families, which inhibit follicular development and prevent ovulation and endometrial receptivity. However, these combined hormone drugs have a serious toll on the health of many women with numerous side effects (Sabatini and Cagiano, 2006; O'Connell et al., 2007) even at lower doses (Rosenberg et al., 1999). Progestin-only contraceptives (“Mini-pills”) reduce many of these risks but are still associated with a high level of discontinuation (McCann and Potter, 1994). There are many reports of the difficulties that women encounter in developing countries to use hormonal contraceptives due to several limitations (Townsend et al., 2011).

Substantial research was, therefore, dedicated to developing non-hormonal contraceptives that could reduce or eliminate side effects. To achieve this purpose, it is vital to identify proteins involved in the process of fertilization, apart from the steroid hormone receptors, so they can be targeted by non-hormonal candidates. Many such proteins were identified over the years, mostly by knock-out experiments or by blocking with antibodies (Gupta et al., 2015). Of those, two emerge as the most crucial ones for initial interaction between gametes: IZUMO1 on sperm, discovered by Inoue et al. (2005) and its oocyte partner, JUNO, discovered by Bianchi et al. (2014). Structures of the JUNO–IZUMO1 complex were published back to back in Nature on 23 June 2016 (Aydin et al., 2016; Ohto et al., 2016). However, more than 5 years later, there has been yet no report of blocking that crucial sperm–egg interaction by small molecules. These structures are the starting points and the only basis for the research presented in this study.

In the present study, we describe a combined effort to discover the blockers of the IZUMO1–JUNO interactions, and beginning with computational predictions of candidate inhibitors of JUNO and testing top candidates by *in vitro* fertilization (IVF) experiments in mice as well as in human sperm–hamster oocytes penetration assay, we found two effective inhibitors of *in vitro* fertilization.

## Results

### JUNO–IZUMO1 Complexes: Most Hot Spots are Common to Both Crystal Structures

**FIGURE 1:**
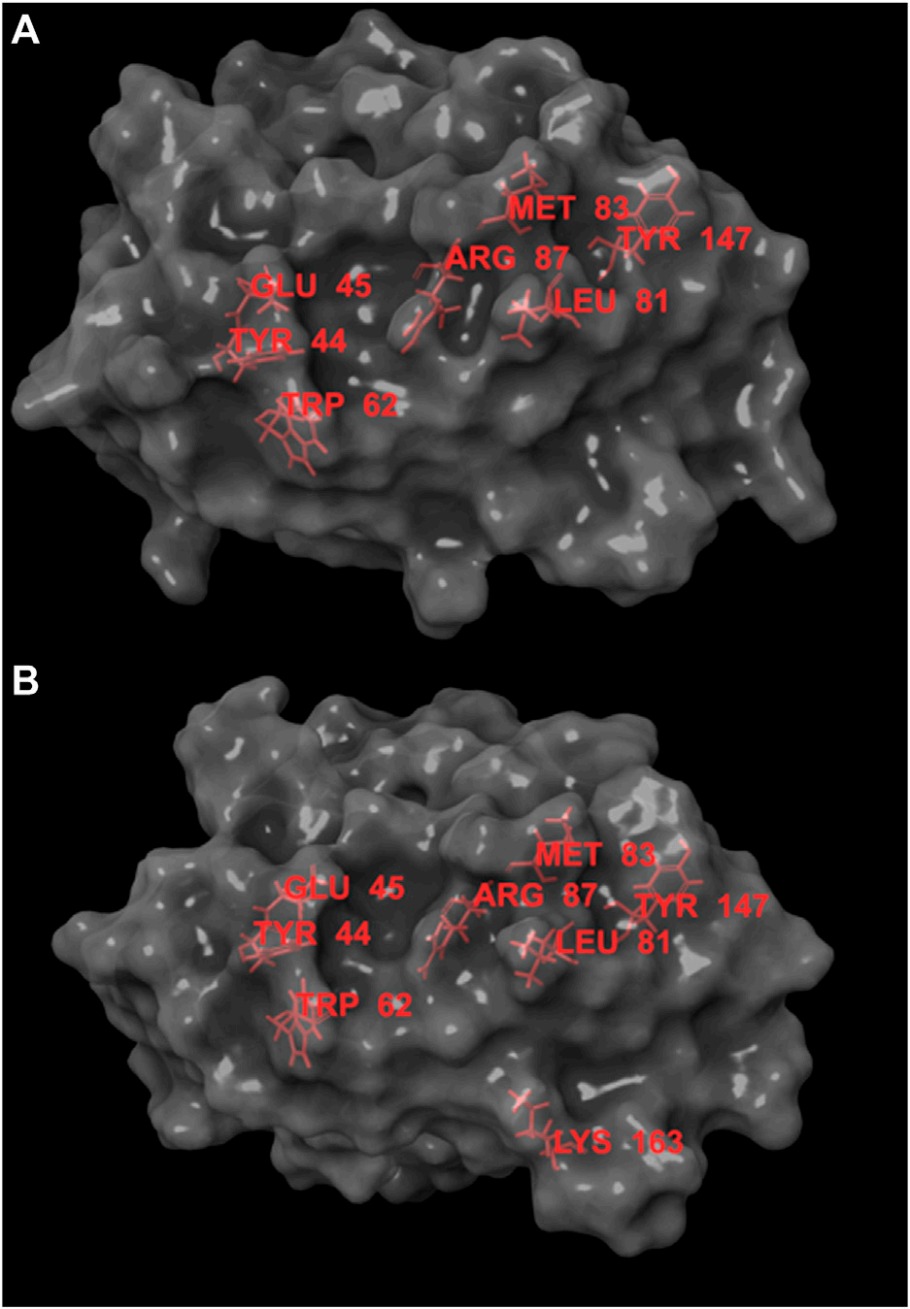
Hot spots for JUNO structures 5JKC **(A)** and 5F4E **(B)**, the hot spots are marked in red. Hot spots are concentrated in an area narrow enough to be covered by molecules with MW ≥ 350. This area contains small grooves that can act as anchor points for ligands and provide partial protection from solvent.

The interface residues of the two JUNO structures, 5JKC and 5F4E, underwent sequential virtual alanine screening by the Bioluminate software (Beard et al., 2013) and the loss of binding energy (delta affinity) was calculated for each virtually mutated residue. The results are listed in Supplementary Table S1 (for 5JKC) and Supplementary Table S2 (for 5F4E). While there are some differences between the two complexes, hot spots (i.e., residues with delta affinity ≥ 4 kcal/mol) were mostly common to both structures. Figure 1 presents the spatial arrangement of hot spots for the JUNO structures.

### Docking Produced Molecules that Interact with Most of the Hot Spots

Following the identification of hot spots, we set the docking site so that it includes most of them and docked 913,000 molecules from the Enamine HTS collection (Enamine Ltd., Ukraine). The molecules were limited to molecular weight greater than 350 g/mol, with the thought that larger molecules have better chances to encompass the full docking site and interact with most of the hot spots. The docked poses were filtered according to the criteria listed in Table 1. These criteria were selected to ensure that the ligands span the entire docking site and interact with most of the hot spots. However, Lys163 and Trp62 were not part of these criteria, Lys163 was excluded as it is too distant from the other hot spots, and Trp62 was excluded as we found that including it resulted in rejection of most molecules from being successfully docked.

**TABLE 1:** Criteria for the filtering of the docked 913,000 molecules from the Enamine HTS collection.

Interaction type	Interacting residue
HBOND	ARG87
Attractive VDW interactions	TYR44, GLU45, MET83, LEU81, TYR147

### Docked Molecules were Selected for the *in vitro* Test by Three Properties

Docked poses that met the aforementioned demands were examined according to the three main criteria: 1) docking score-the main energy parameter used in GLIDE (Friesner et al., 2004), 2) Attractive VDW contacts-number of Van der Waals contacts favorable for ligand affinity, and 3) BSA (buried surface area)- a rough measure of a favorable hydrophobic contribution to the entropy.

**FIGURE 2:**
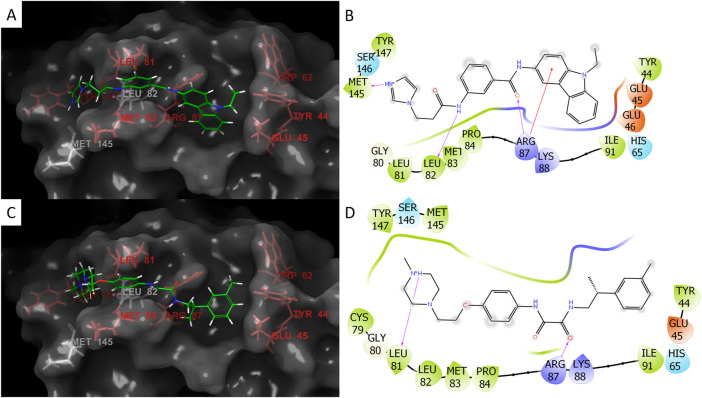
Docking poses and interactions of the two inhibitors Z786028994 **(A,B)** and Z1290281203 **(C,D)**. For the 3D images of the poses: hot spots- red, non-hot spot residues- white, and ligand- green. For the 2D interaction plots: negative charge- red, positive charge- blue, hydrophobic residues- green, hydrogen bonds- purple arrows (the direction is donor to acceptor), and red line to the aromatic ring represents Pi-cation interaction. Both molecules span the length of the docking site and the small grooves in the protein provide partial protection from solvent **(A,C)**. Z786028994 has hydrogen bonds with ARG87, LEU82, and MET145 **(B)** and Z1290281203 has hydrogen bonds with two hot spots, ARG87 and LEU82 **(D)**.

We used these three properties to rank the molecules and to select several molecules for *in vitro* test. These molecules are listed in Table 2. Ideally, ligands should meet the geometric criteria in full and have better docking score and maximal VDW contacts and BSA. However, in some instances compromises were made to accommodate for molecules we deemed promising by viewing. For example, the molecule Z1172207733 produced a mediocre docking score, but had a large BSA that may result in a gain of entropy sufficient to cause the ligand to effectively bind to JUNO. On the other hand, the molecule Z49734016 only partially met the geometric criteria as it did not interact with TYR44 or TYR147. However, it had a very negative docking score, and a large number of VDW contacts, and so it was also picked for testing. Most of the molecules that were sent for experimental validations are from docking to 5JKC, apart from Z66693270 that was docked to 5F4E. Two of these molecules, Z786028994 and Z1290281203, were found to block *in vitro* fertilization in mice (described below), and their poses and interaction patterns with the JUNO residues are shown in Figure 2. Both inhibitors have VDW interactions with all of the hot spots, as well as a hydrogen bond with ARG87. In addition, Z1290281203 has a hydrogen bond with LEU81, and Z786028994 has hydrogen bonds with LEU82 and MET145.

**TABLE 2:** Top scored molecules sent for the *in vitro* test.

Molecule ID	Docking score (Kcal/mole)	Number of attractive VDW contacts	BSA (Å^2^)
Z49720304	−4	292	843
Z18823321	−3.8	233	883
Z1290281203	−3.4	272	848
Z1033235866	−3.4	220	751
Z131775002	−4.4	211	748
Z56788505	−5.4	316	1,039
Z786028994	−4.3	241	832
Z1172207733	−2.4	293	986
Z49734016	−7	453	779
Z66693270	−3.3	210	818

### ISE Model can Differentiate Between the “Best” and “Worst” Docked Molecules

**FIGURE 3:**
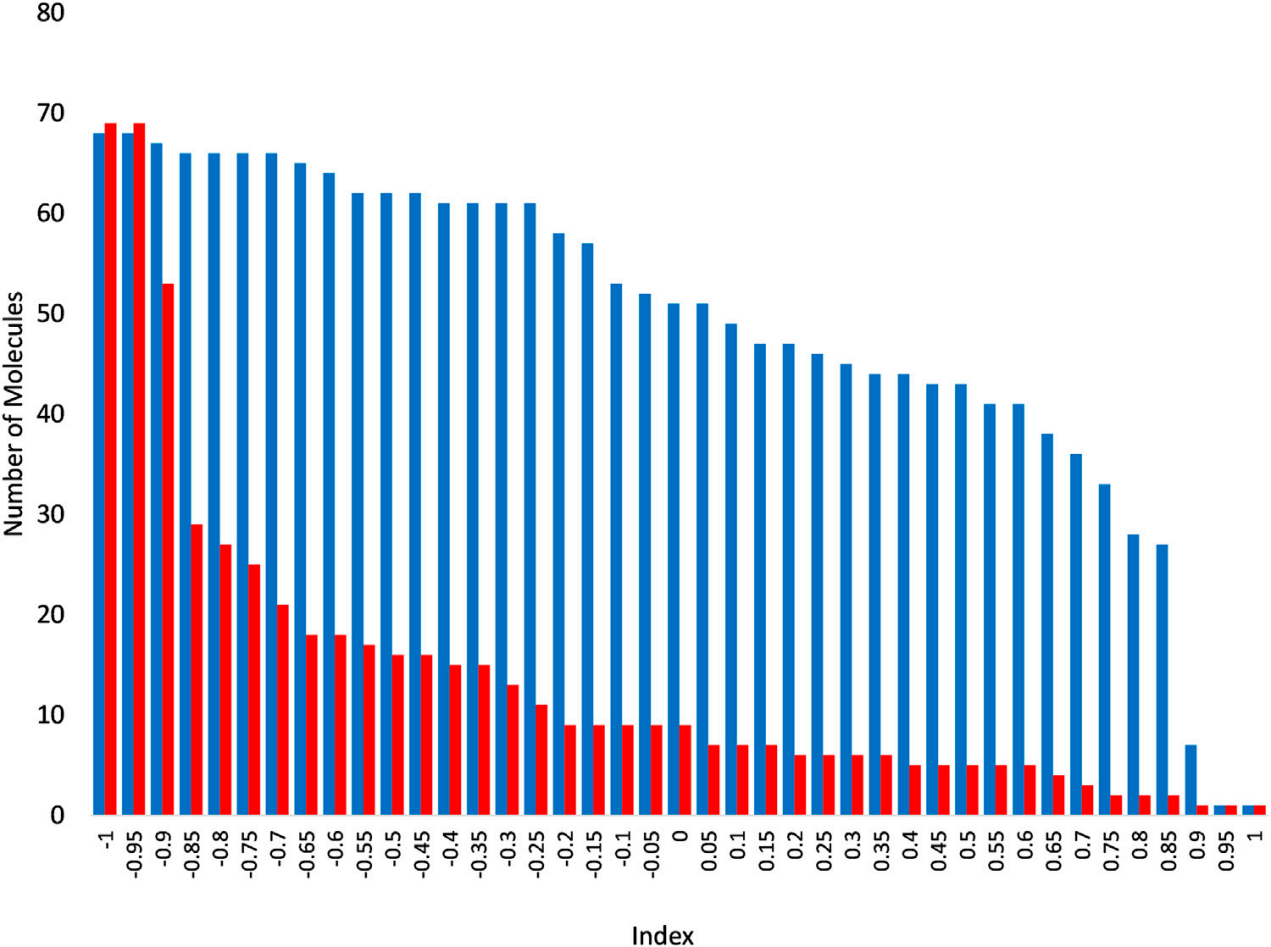
Numbers of “Best” (blue) and “Worst” (red) above each index following screening through the ISE model. As the index increases, the number of molecules decreases in both the “Best” and “Worst” molecular sets, but at a much slower rate for the former than the latter, and so the remaining set is increasingly enriched with “Best” molecules.

In order to expand the pool of candidate molecules, a classification model was constructed using the Iterative Stochastic Elimination (ISE) algorithm (Stern and Goldblum, 2014) and was based on the docking results to the 5JKC JUNO structure: Docked molecules that met the geometric criteria were divided into 68 “Best” molecules (top quartile) and 69 “Worst” molecules (bottom quartile) according to the criteria listed in Table 3. The two sets were combined into a learning set, for which 206 molecular descriptors were calculated by MOE2018.0101 [Molecular Operating Environment (MOE) and Chemical Computing Group ULC, 2018]. The learning set was randomly divided into five parts or “folds”, each containing 20% of the “Best” and 20% of the “Worst” molecules. Each four folds in turn were combined into a training set, to which the ISE algorithm was applied and produced a set of filters, and the remaining fold (i.e., the test set) was screened through those filters and its molecules were scored. Due to the iterations of different folds, all molecules were evaluated as part of a test set in one of the five different runs of modeling which were combined to produce a single, final model. .The final model consisted of 919 filters with good statistical criteria, mainly the Matthews Correlation Coefficient [MCC, (Stern and Goldblum, 2014)] values ranging from 0.86 to 0.77 for the top and bottom filter, respectively. Screening of the molecules in the test sets produced molecular indexes between −1 and 1, indicating a success or failure to pass the filters. The numbers of “Best” and “Worst” molecules at or above each index are shown in Figure 3. As the index increased so did the ratio of true to false positives (TP/FP), from one at an index of −1 up to 16.5 at an index of 0.75. That index was chosen to be the cutoff for candidate selection from the entire dataset, in order to minimize the number of false positives. The Enamine HTS collection of ∼1.8 million molecules (Enamine Ltd., Ukraine) and a dataset of ∼20 million molecules from the ZINC database (Sterling and Irwin, 2015) were screened through the model, out of which 31,555 molecules scored at or surpassed that cutoff index of 0.75. These molecules were subsequently docked to 5JKC and 5F4E using the SP method of Schrodinger’s Glide (Friesner et al., 2004).

**TABLE 3:** Criteria for best and worst docked molecules.

Criteria	Best	Worst
Docking score	< −3	≥ −1
BSA	≥750	≤600
Number of VDW contacts	≥250	≤150

We wished to examine whether interaction with only a part of the hot spots may be sufficient for blocking the JUNO–IZUMO1 interaction, and so, in addition to candidate molecules that span across the entire site (as detailed in Table 3), we divided the docking site into two sub-sites as listed in Table 4, and included molecules that interact with the hot spots on either one of those sub-sites. Here, we could include TRP62 in the criteria for successful docking to the sub-site B, as molecules docked to that site were sufficiently close to it. LYS163, on the other hand, remains too distant for most molecules to interact with. The 331 molecules that passed the filter were ranked by the three properties mentioned above, and 22 top scored molecules were sent for *in vitro* test.

**TABLE 4:** Criteria for the filtering of the docked 31,555 molecules from the ISE model.

Interaction type	Sub-site A	Sub-site B
HBOND	ARG87	ARG87
Attractive VDW interactions	MET83, LEU81, TYR147	TYR44, GLU45, TRP62

### Z786028994 and Z1290281203 are Potent Fertilization Inhibitors

**FIGURE 4:**
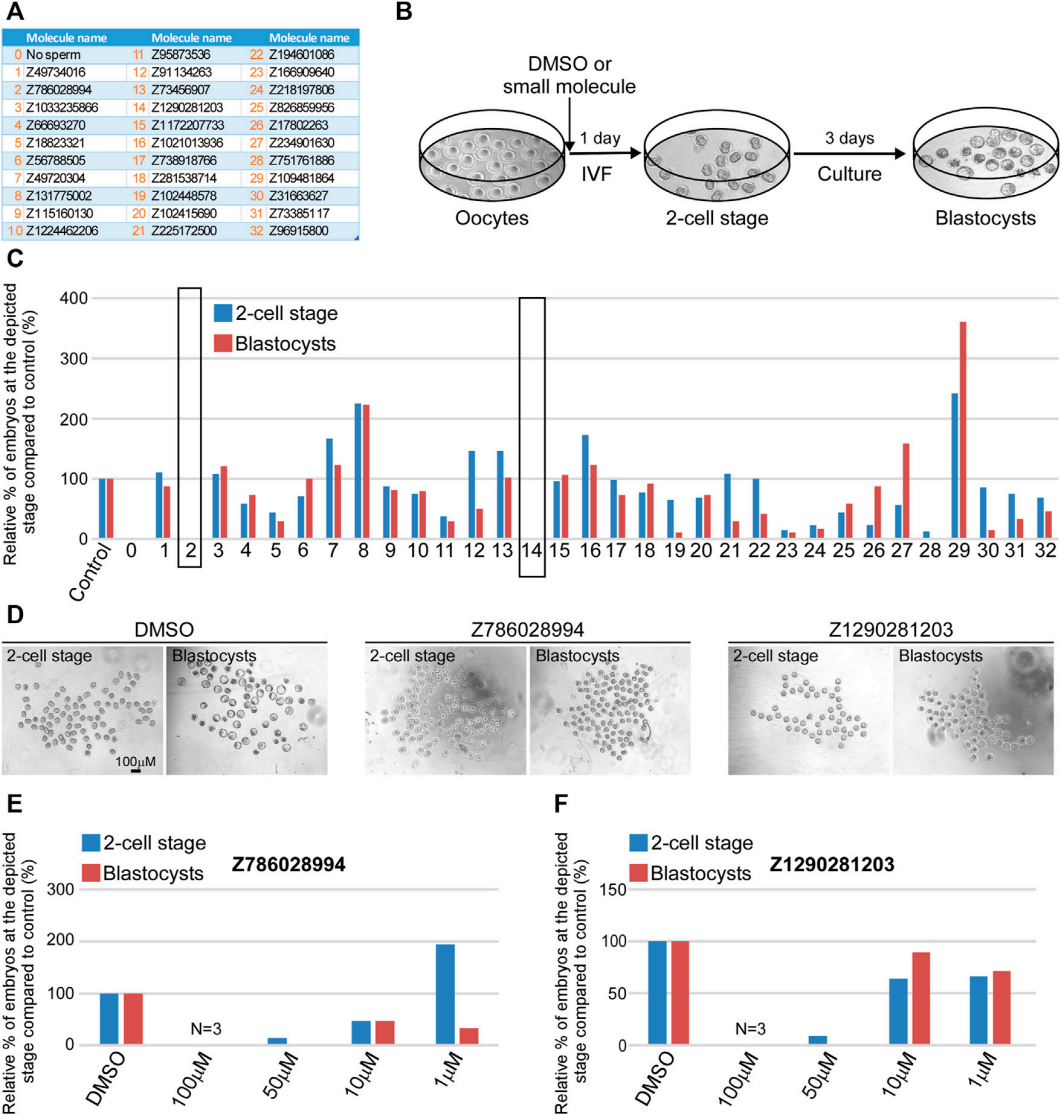
Z786028994 and Z1290281203 demonstrate fertilization blockage at a concentration of 100 and 50 μM. **(A)** A table depicting a list of small molecules that were predicted to block JUNO–IZUMO1 interaction and were tested using IVF. **(B)** An illustration of the IVF experimental setup scheme showing the progression of embryo development after IVF. At day 1, the fertilized eggs developed into 2-cell stage embryos following by blastocysts formation at day 4 of fertilization. **(C)** Relative percentage of live embryos at day 1 (2-cell stage) and 4 (blastocyst stage) compared to positive (DMSO) and negative (no sperm) controls. All molecules presented in the graph were tested at a concentration of 100 µM. Small molecules Z786028994 and Z1290281203 (marked by black rectangle) demonstrated a complete blockage of fertilization, showing only unfertilized oocytes at day 4. **(D)** Representative bright field images of the developing embryos at day 1 (2-cell stage) and 4 (blastocyst stage) post IVF treatment in control (DMSO) and when the two small molecules were added to the extracted oocytes prior to sperm addition. **(E)** Relative percentage of the embryos at the depicted stages in the presence of different concentrations of the molecule Z786028994 compared to the DMSO control. **(F)** Relative percentage of the embryos at the depicted stages in the presence of different concentrations of the molecule Z1290281203 compared to the DMSO control.

Our *in silico* analyses identified 32 small molecule candidates (Figure 4A) that were predicted to bind to JUNO and could block its interaction with the sperm receptor IZUMO1. One stringent test to assess fertilization blockage is *in vitro* fertilization (IVF) assay (Figure 4B). Thus, we next employed IVF to test the inhibitory effect of the 32 (10 from structure based docking, 22 from ISE ligand based modeling) small molecule candidates on fertilization. To that end, we extracted oocytes from superovulated female mice and placed them in a dish containing 100 μM of either DMSO control or small molecule candidates. Immediately after, activated sperm were added to the plate and the formation of 2-cell stage embryos and blastocysts was scored after one and 4 days post sperm addition. The results of all experiments performed are detailed in Supplementary Table S3 and shown in Figure 4. The vast majority of the tested molecules did not show any significant fertilization inhibition neither at the 2-cell stage nor on blastocyst formation (Figure 4C). Remarkably however, two small molecules namely, Z786028994 and Z1290281203 (#2 and #14, marked by black rectangle), showed no formation of either 2-cell stage embryos or blastocysts, and the small molecule Z751761886 (#28) demonstrated very few 2-cell stage embryos (Figure 4C). Importantly however, in contrast to Z751761886, the blockage seen with Z786028994 and Z1290281203 was not due to a toxic effect as the oocytes remained healthy even after 4 days of culturing (Figure 4D). Moreover, the accumulation of sperm cells in the perivitelline space of eggs treated with molecules Z786028994 and Z1290281203 strongly suggests that the fertilization failure seen by these two molecules is a result of inhibition of sperm–egg fusion (Supplementary Figure S1). We then moved on and tested those two molecules using lower concentrations. While a concentration of 50 µM still exhibited a strong inhibitory effect, yielding only few 2-cell stage embryos with zero blastocyst formation after 4 days of culturing, at lower concentrations of 10 and 1 μM no significant inhibitory effect was seen with these two molecules (Figures 4E,F). Taken together, these results indicate that Z786028994 and Z1290281203 are potent fertilization inhibitors at a concentration of 100 and 50 µM and suggest that further exploration of these small molecules is required to identify derivatives that might be able to block fertilization even at lower concentrations.

### Z786028994 had the Largest Inhibition Effect on Human Sperm Fertilization in Hamster Penetration Assay

**FIGURE 5:**
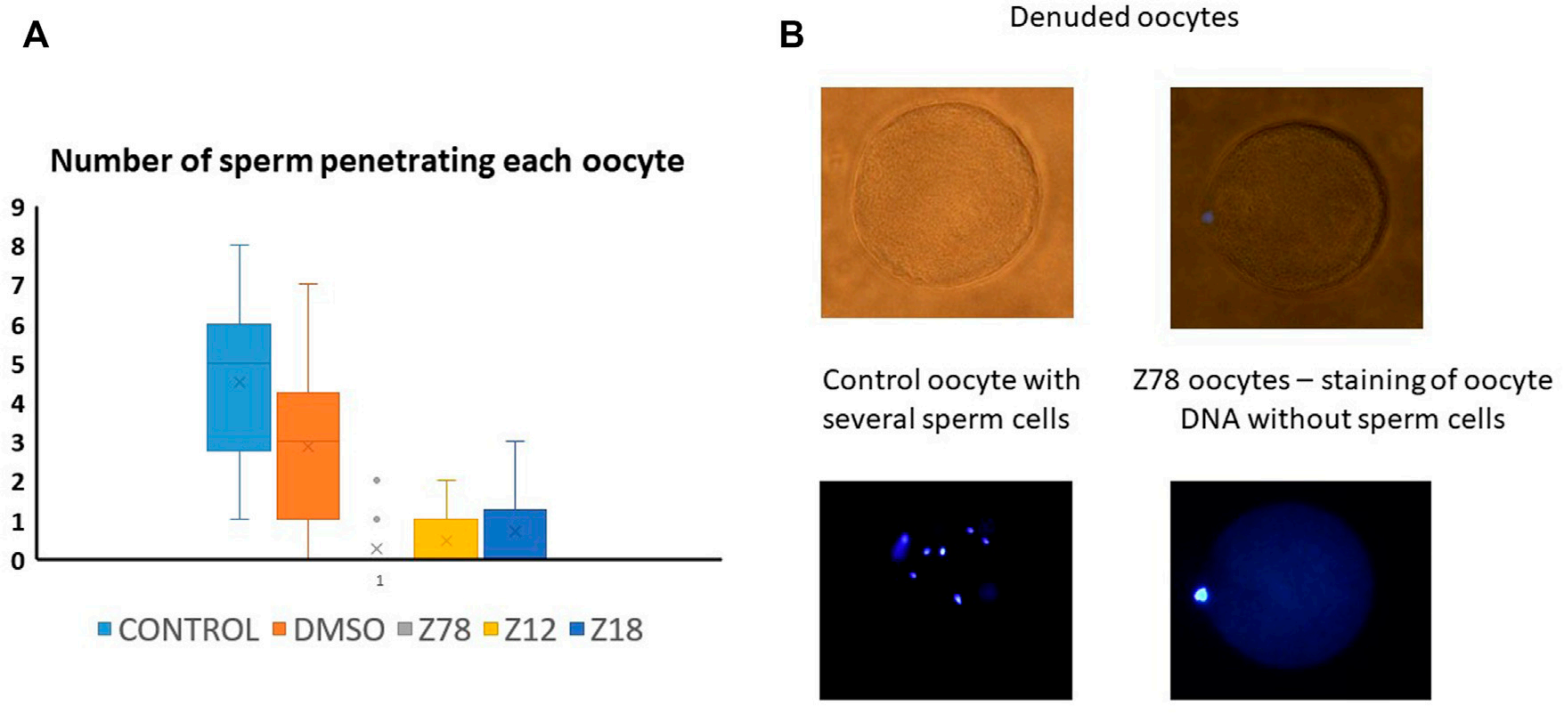
Z786028994, Z1290281203, and Z1290281203 (marked Z78, Z12, and Z18) demonstrated penetration blockage at a concentration of 100 μM. **(A)** Number of penetrated human sperm cells into denuded hamster oocyte compared to control and DMSO. All molecules presented in the graph were tested at a concentration of 100 µM. The three tested molecules demonstrated inhibition of penetration, with the most potent inhibition by Z786028994 molecule. **(B)** Representative bright field and DAPI images of oocytes after penetration assay. Several sperm cells demonstrated inside the oocytes in the control group compared to no sperm cells penetrating the oocytes when incubated with Z786028994 molecule (just staining of the polar body).

We picked the two most effective inhibitors for the hamster penetration assay from the mice experiment (Z786028994 and Z1290281203). In addition, we chose Z18823321 as being a less effective inhibitor (Figure 4). Following co-incubation with sperm, Z786028994 had an inhibitory effect on sperm and oocytes fusion, with a statistically significant difference in the number of penetrated sperm when compared either to the DMSO or to the control (Figure 5). The two other molecules (Z1290281203 and Z18823321) displayed a somewhat less inhibitory effect while having a statistically significant effect compared to DMSO. As motile sperm were observed after incubation with the molecules, the inhibition was not ascribed to a molecular toxic character. We further excluded an effect of the DMSO on sperm penetration by demonstrating no statistically significant difference in the number of penetrated sperm when incubated sperm with the DMSO alone was compared to the control.

### The Small-Molecule Candidates Fail to Demonstrate JUNO–IZUMO1 Binding Interference using AVEXIS

**FIGURE 6:**
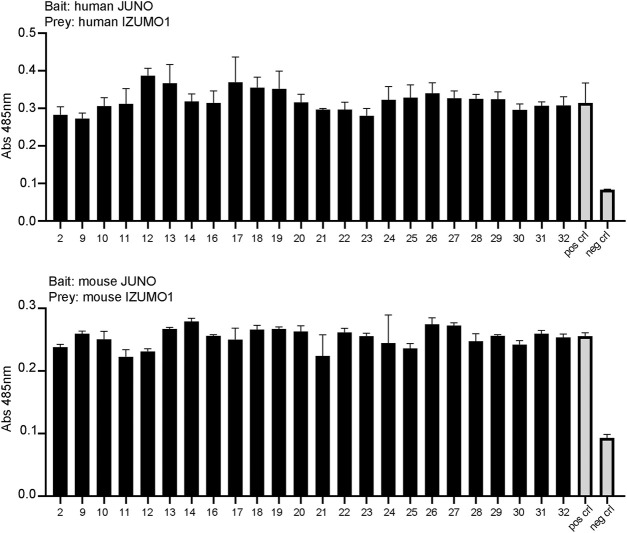
The small molecules tested by AVEXIS did not show the ability to interfere with JUNO–IZUMO1 binding. The soluble ectodomains of JUNO and IZUMO1 were expressed as biotinylated bait and pentameric preys, respectively. Biotinylated JUNO was immobilized on a streptavidin-coated plate and probed for the ability to bind the beta-lactamase-tagged prey IZUMO1. The candidate molecules were incubated with the baits and with the preys at a concentration of 100 μM while DMSO was added to positive and negative controls. The same concentration was maintained in all steps of the assay. The binding of baits and preys was detected by the enzymatic turnover of a colorimetric substrate and quantified by absorbance readings. None of the small molecules significantly affected the binding of JUNO and IZUMO as shown by the AVEXIS performed with human **(upper panel)** and mouse **(lower panel)** proteins.

To assess whether the small molecules could prevent the binding of JUNO and IZUMO1, we used an *in vitro* assay specifically developed to detect the interaction of receptor ectodomains named AVidity-based Extracellular Interaction Screening (AVEXIS) (Bushell et al., 2008). The ectodomain of JUNO was expressed as a soluble recombinant bait, captured on a solid surface and probed for its ability to bind the soluble pentameric ectodomain of IZUMO1. The candidate molecules or DMSO were incubated with both JUNO and IZUMO1 for 30 min, and then maintained in the media throughout the assay. Murine and human proteins were tested separately due to the species-specific characteristics of the binding (Bianchi and Wright, 2015). None of the molecules significantly affected the binding of JUNO and IZUMO1 (Figure 6).

## Discussion

The structure of the JUNO–IZUMO1 complex was elucidated by X-ray crystallography more than 5 years ago and published back to back in Nature, by two different groups. Ohto et al. wrote that “IZUMO1 and JUNO are ideal targets for contraceptive agents because of their crucial involvement in fertilization”. Aydin et al. suggested “promising benefits for the rational development of non-hormonal contraceptives and fertility treatments for humans and other mammals”. It should therefore be extremely surprising to learn that these possibilities did not produce any published echo in the research community. Until today, there are no public reports on finding of any novel inhibitor of fertilization by blocking the JUNO–IZUMO1 interaction.

In the absence of known inhibitor structure, it is natural to use the known complexes in order to try to mimic one partner’s interactions with the other partner. There are only two alternatives: either block JUNO or block IZUMO1. We decided on JUNO due to several advantages such as location (on the Egg’s surface), surface structure (druggability due to surface crevices), relative conformational rigidity (compared to the large change in IZUMO1’s conformation between free and bound states), and a somewhat smaller size of JUNO.

We used the structure of the JUNO–IZUMO1 complex in order to locate the most crucial (hot spots) JUNO residues for binding IZUMO1. By using virtual alanine scan—replacing each residue of JUNO that interacts directly with IZUMO1, we found seven such potential hot spots that occupy a large region on the surface of JUNO. The interaction energy difference due to mutations of these residues to alanine may be different in computations than by *in vitro* alanine scan. *In vitro*, the protein can change conformation due to replacing a side chain by the CH3 of alanine (except for Gly) and that change has an energy component. In the computations, we modify only a specific side chain but do not allow any conformational change (which may be achieved by minimizations or by Molecular Dynamics). The idea behind that restriction is that we wish to replace the interactions of IZUMO1 with JUNO in exactly the same structure that is reported in the Protein Data Bank. We do not allow rotations around the Cb-Ca bond of alanine, assuming that it has only a minor effect on enthalpy and entropy of the side chain replacement.

We Docked 913,000 molecules to the hot spot regions of 5F4E and 5JKC. We then filtered the docked poses and included those that interact with most of the hot spots, and ranked them by their docking scores, VDW contacts, and BSA. Buried surface area was considered for its contribution to the translational and rotational entropy due to the release of water molecules from the binding site to the bulk solution, a factor which is lacking in docking score calculations. Ten candidates from the top ranked molecules were sent for IVF experiments. Two molecules Z786028994 and Z1290281203 were found to fully inhibit *in vitro* fertilization in mice with concentrations of 50 and 100 μM, while some of them had a partial effect (like Z18823321). Z786028994 also completely blocked human sperm penetration to the hamster oocytes, while Z1290281203 and Z18823321 blocked it partially. Following a reviewer’s comment that sperm swelling is a standard indicator of sperm penetration, we consider our results to be indicative at least of sperm oolema adhesion, being totally different for Z786028994, Z1290281203, and Z18823321 than for the DMSO or control. That is also corroborated by the fact that all samples were similarly treated by washing.

The docked poses of full inhibitors in mice, Z786028994 and Z1290281203, indicate that they interact with all the hot spots via VDW interactions. In addition, Z1290281203 has hydrogen bonds with two hot spots, ARG87 and LEU82, and Z786028994 has hydrogen bonds with ARG87, LEU82, and MET145 (Figure 2). The two latter residues are not hot spots, however, those hydrogen bonds may help stabilize the molecule in the specific pose. If confirmed by structural studies in crystals, the interactions of these two inhibitors can support pharmacophore-based virtual screening that would help in finding additional blockers of IVF.

Discovering two inhibitors out of ten candidates from docking less than a million molecules could suggest that docking a much larger number of molecules could find additional ones. We have only recently added nearly 200 million molecules to our “arsenal” of molecules, with about 20 million molecules from ZINC (Sterling and Irwin, 2015) and about 160 million molecules from a recently published ultra-large docking library (Lyu et al., 2019). With such proportion of success, we could envisage many more successful candidates out of that large set of candidates for docking.

Docking of databases containing millions of molecules is extremely time consuming. To allow us to exploit such large databases, we attempted to use the results of docking of the Enamine database described previously for ligand-based classification modeling. The two classes we used are the set of top third scored and bottom third scored molecules. Those were defined as “Best” and “Worst” classes to produce an ISE model that successfully discriminated between them. Screening of ∼22 million molecules by that model found a large number of candidates with high scores that were docked, filtered, and ranked as mentioned above, and 22 top ranked molecules were sent for IVF tests. Unfortunately, none of these predicted molecules blocked IVF. While ISE has an excellent track record of finding novel molecules with a desired activity or property, including low micromolar hits and nanomolar leads (Cern et al., 2017; El-Atawneh and Goldblum, 2017; Da’adoosh et al., 2019; El-Atawneh et al., 2019; Da’adoosh et al., 2020), in the present case it has failed to do so. The successful ISE models were trained using published experimental data, in which the number of false positives is expected to be small. In the present case, in contrast, we used computed docking results to replace the non-existing experiments. Out of ten molecules found by docking, only two were confirmed hits. Assuming this proportion is representative for all our docking results, that means that 80% of the “Best” set are probably false positives of the docking method. Thus, it is likely that this set cannot be used to train models to identify IVF blockers.

Two separate IVF experiments were used to confirm the inhibitory activity: mouse sperm–mouse oocytes and human sperm–hamster oocytes, combined with the results of computational docking, suggest that our compounds indeed block JUNO on the oocytes of both species. However, all attempts to discover such inhibition by the AVEXIS technique developed by Bianchi and Wright did not produce evidence for direct inhibition. The direct interaction experiments were performed with HEK293 cells rather than oocytes, and it is possible that there are factors in oocytes’ membrane environment (such as other membrane proteins) that interact with JUNO and stabilize it in a conformation susceptible to the binding of the inhibitors prior to contact with the sperm, and so they are in place to disrupt the interaction with IZUMO1 and block subsequent fertilization. It should also be noted that the AVEXIS assay is specifically designed to detect the interactions between ectodomains, and it is possible that the inhibitors may disrupt some other essential function of IZUMO1 or JUNO other than its extracellular binding activity such as JUNO shedding (Bianchi et al., 2014) or structural rearrangements of IZUMO1 upon JUNO binding to bind other but as yet unidentified egg receptors (Inoue et al., 2015). However, until further research is performed, the mechanism of action of these IVF inhibitors remains unclear.

## Methods

### Prediction of Hot Spots

Two crystal structures of a JUNO–IZUMO1 complex are our basis for searching to discover non-hormonal contraceptives (PDB codes 5JKC and 5F4E) (Aydin et al., 2016; Ohto et al., 2016). Both structures were prepared for docking using mostly the default settings of the protein preparation wizard of the Schrödinger software 2018-4 release (Schrödinger, 2021), with the exception of the minimization stage which was performed only on the computationally added protons. The JUNO interface residues involved in the binding of IZUMO1 were extracted from the PDBsum website (Laskowski et al., 2018). Each of these residues was virtually mutated to alanine in its turn using Schrödinger’s Bioluminate software 2018-4 release (Beard et al., 2013). Following each mutation, the loss of binding energy was calculated. Mutated residues with an energy loss of 4 kcal/mol or more were considered to be hot spots.

### Docking and Pose Filtering

The docking sites were set by GLIDE’s (Friesner et al., 2004) grid generation to encompass the hot spots of the JUNO structure. Molecules were prepared using the default settings of the Ligprep panel in the Schrödinger software 2018-4 release, and docked to the JUNO structures using Glide’s fast High Throughput Virtual Screening (HTVS) method. The resulting poses were then filtered by a set of geometric criteria that use the JUNO hot spots in order to maximize the competitive nature of potential inhibitors versus JUNO–IZUMO1 interactions. Molecules that met these criteria were re-docked using the slower standard precision (SP) method and rechecked for matching the geometric criteria. The molecules were then ranked according to three properties: docking score, number of attractive Van der Waals contacts with JUNO, and the buried surface area (BSA) created by the ligand–JUNO interaction. Top ranked molecules were sent for *in vitro* test.

### Construction of the ISE Models

The Iterative Stochastic Elimination (ISE) algorithm had been previously described in detail (Stern and Goldblum, 2014). It has been successfully used by us to discover novel active molecules, based on an initial set of known activities of a set of molecules. Models were built by distinguishing between properties of these actives (usually >50 molecules) and properties of a large group (diluting the actives by 100 fold or more) of randomly picked “decoys” that represent “chemical space”. A tougher case for successful classification is to distinguish among the actives, between highly active and less active molecules, usually separated by one or two orders of activity values and having a comparable set size. Once a model is achieved and is justified by statistics, we screen millions of molecules by that model and score each of them, subsequently picking the top ones which may be docked to their target if its structure has been elucidated. The problem with JUNO was and still is, 7 years after its discovery (Bianchi et al., 2014) that there are no known small molecule inhibitors of JUNO. We have thus decided to use the results of docking as if they were experimentally validated.

Docking results were thus divided into two sets of molecules “Best” and “Worst”, according to the criteria based on the three properties mentioned above. For both these sets, 206 molecular descriptors were calculated by MOE2018.0101 (Molecular Operating Environment (MOE) and Chemical Computing Group ULC, 2018) and were then unified into a single learning set. All further steps in applying the ISE algorithm to results of docking are similar to any application of ISE as reviewed in Stern and Goldblum (2014).

### Preparation of IVF Medium and Fertilization Drops

All powders listed in Supplementary Table S4 were weighted as indicated and diluted in water for embryo transfer (Sigma, W1503). The medium was filtered through 0.22 µm filter and stored at 4°C for up to 3 months. At the morning of the IVF experiments, 30.7 mg of reduced glutathione (GSH) was added to 1 ml of HTF and mixed. 50 µL from this solution was added to the 5 ml of fresh HTF medium, filtered and used to prepare fertilization dishes. 90 µL drops of HTF medium with GSH were placed on the bottom of 35 mm Petri dishes (Falcon 351008), covered with mineral oil and incubated at 37°C, 5% CO_2_ for 20 min.

### Preparation of TYH + MBCD Sperm Pre-Incubation Medium

All powders listed in Supplementary Table S5 were weighted and diluted in water for embryo transfer (Sigma, W1503). The medium was filtered through a 0.22 µm filter and stored at 4°C for up to 3 months. At the morning of IVF experiments, 180 µL drop of pre-incubation medium was placed on the bottom of 35 mm Petri dishes (Falcon 351008) and covered with mineral oil.

### Preparation of Small Molecules

Inhibitors were purchased from Enamine (Enamine Ltd, Ukrain), diluted in DMSO to a stock concentration of 10 mM and stored at −80°C. Each diluted inhibitor (100 µM or as indicated) was added to the HTF medium with GSH prior to sperm addition. The fertilization drops with each molecule was carefully marked, placed in humidity incubator at 37°C, 5% CO_2_ for 30 min, and used for IVF.

### Superovulation of the Female Mice

For each independent experiment four female mice, 3 weeks old, strain CB6/F1 were injected intraperitoneally (IP) with PMSG hormone (ProSpec, hor-272-a), and 48 h later with hCG (ProSpec, hor-007) hormone, 5IU per animal of average weight of 20 g.

### Preparation of Capacitated Sperm

A male mouse of reproductive age was placed in an individual cage for 3 days before IVF experiments. On the morning of the IVF, the cauda, a structure located below the testis, was extracted and placed into a drop of TYM medium + MBCD. Few excisions were made to allow the sperm migrate out of the cauda to the medium. The plates were placed in humidity incubator at 37°C, 5% CO_2_ for 1 h and stirred gently every 15 min. Following this incubation, activated sperm (sperm along the edges of the drop) were used for IVF.

### *In vitro* Fertilization

The oviducts of superovulated mice were extracted 13.5 h after hCG injection and placed into a pre-warm M2 medium. The ampulla was tore with a needle and the oocytes were transferred into fertilization drops containing HTF medium with GSH and 100 µM of inhibitor candidate. Drops containing DMSO were used as control for all inhibitors. Activated mouse sperm were immediately added to the fertilization drops and incubated at 37°C, 5% CO_2_. Following 3–4 h of incubation the eggs from each group (control and experiment) were washed with few drops of fresh HTF medium without GSH to remove cell debris, degenerating oocytes, and dead sperm. The next morning the dishes were screened under the microscope and the percentage of 2-cell stage embryos was assessed in the control and in the experimental groups. The counting was repeated at day 4 to assess the percentage of developed blastocysts.

### Hamster Oocyte Retrieval

Mature female golden hamsters 8–12 weeks old were injected with 30 IU of pregnant mare serum gonadotropin (PMSG, ProSpec-Tany TechnoGene Ltd., Ness-Ziona, Israel) intraperitoneally, followed by an injection of 37 IU of human chorionic gonadotropin (hCG, ProSpec-Tany TechnoGene Ltd., Ness-Ziona, Israel) 56 h later. The hamsters were euthanized using a CO_2_ chamber 17 h after administration of hCG injection. The oviducts were excised and placed in culture dishes containing saline. The ampule was tore and cumuli-oocyte complexes were collected and treated with hyaluronidase (SAGE, Trumbull, United States) for 1–2 min of incubation at 37°C. The remained cumulus cells were mechanically denuded with stripped pipette in 10% HEPES (SAGE, Trumbull, United States).

### Hamster Penetration Assay

Human donor sperm were accepted from the sperm bank at the Hadassah Medical Center. The donors signed in advance with consent to use the sperm for donation or for research (approved by the local IRB). The frozen sperm dissolved on room temperature, loaded on gradient centrifuge, and washed with Multipurpose Handling Medium-Complete (Fujifilm Irvine Scientific Inc., Santa Ana, CA, United States). Dissolution of the zona pellucida was performed by placing the oocytes in 0.5 mg/ml trypsin (Biological industries, Israel) for 8 min. The oocytes were evenly divided and were incubated in 10% fertilization medium (SAGE, Trumbull, United States) with either Z786028994 (100 µM), Z1290281203 (100 µM), Z18823321 (100 µM), DMSO or no addition as control for 1 h at 37°c and 5% CO_2_. The oocytes were then transferred into 100 µL drops with 250,000 motile human sperm with the same concentration of the inhibitory molecules (as mentioned above) and incubated at 37°C for 3 h. Finally, the oocytes were rinsed from extra sperm cells, fixed with formaldehyde, and stained with DAPI. The slides were examined under a fluorescence microscope at a 400 × magnification. The number of penetrated sperm in each oocyte was recorded.

### Protein Expression and Interaction Screening by AVEXIS

All proteins were produced by transient transfection using an HEK293-6E expression system, and the cells were transfected with 1 μg/ml of plasmid DNA. The cells were incubated for 5 days in a shaking incubator at 37°C before supernatants were harvested, the cells were removed by centrifugation at 3220 g for 20 min, and the cell debris was removed by filtration.

Bait and prey proteins were normalized to activities that have been shown to detect transient interactions and screened using the ELISA-based AVEXIS methodology essentially as described (Kerr and Wright, 2012). 100 μL of biotinylated baits were immobilized on streptavidin-coated 96-well microtitre plates (Greiner) and washed with HBS 0.1%Tween. After washing 100 μL of normalized β-lactamase-tagged preys were added and incubated for 1 hour at room temperature. The wells were washed with HBS 0.1%Tween and finally 125 μg/ml of the β-lactamase substrate (Nitrocefin) was added. Absorbance values were measured at 485 nm on a Spark (Tecan) plate reader. The assays were repeated three times using independent protein preparations.

Biotinylated JUNO proteins were used as baits, while pentameric IZUMO1 proteins were used as preys. The small molecules were dissolved in DMSO at a concentration of 10 mM and diluted 100 times into the protein supernatants 30 min before they were added to the plates. JUNO and IZUMO with 1% DMSO were used as the positive control; the biotinylated extracellular fragment d3d4 of the rat CD4 was incubated with pentameric IZUMO1 and used as the negative control.

## Data Availability

The original contributions presented in the study are included in the article/Supplementary Material, further inquiries can be directed to the corresponding author.
