# Supplementary material for: In silico Docking Analysis for Blocking JUNO‐IZUMO1 Interaction Identifies Two Small Molecules that Block in vitro Fertilization
Source: Front Cell Dev Biol. 2022 Apr 5;10:824629. doi: 10.3389/fcell.2022.824629 (PMC9037035; doi:10.3389/fcell.2022.824629)
Supplement: Supplementary file 1 [file DataSheet1.docx]

**Supplementary materials**

Five tables of which the first two –Table S1 and S2 report the hot spot residues in the two crystal structures 5JKC and 5F4E, Table S3 details the IVF developmental rate following small molecule treatments, Tables S4 present the composition of high calcium HTF media and Table S5 presents the high calcium TYH+MBCD sperm pre-incubation medium.

Figure S1 Displays the accumulation of sperm at the perivitelline space of Z786028994 and Z1290281203 treated oocytes

| **Table S1. Hot spot residues for the JUNO structure 5JKC and their interactions with IZUMO1** | | | | |
| --- | --- | --- | --- | --- |
| **JUNO residue** | **Δ Affinity, kcal/mole** | **IZUMO1 residues with VDW interactions < 4 Å** | **Additional Interactions** | **IZUMO1 Residue** |
| TYR44 | 14.2 | SER241, ARG160, VAL141 | H-bond, Oh | ARG160, Nh2 |
| GLU45 | 4.4 | SER241, SER240, ASN239 | H-bond, Oe1 | SER240, N |
|  |  |  | salt bridge, Oe2 | ARG160, Nh2 |
| TRP62 | 19.9 | LYS161, TYR163, SER162, ARG160 |  |  |
| LEU81 | 10.3 | TRP148, LYS150, MET75, TYR134, VAL77, LEU146 | H-bond, O | TRP148, Ne1 |
|  |  |  | H-bond, O | LYS150, Nz |
|  |  |  | H-bond, N | MET75, O |
| MET83 | 14.4 | ASN151, GLU155, LYS153, TRP148 |  |  |
| ARG87 | 11.3 | ALA158, HIS157, ARG160 | H-bond, Nh2 | ALA158, O |
| TYR147 | 10.7 | LYS150 |  |  |

| **Table S2. Hot spot residues for the JUNO structure 5F4E and their interactions with IZUMO1** | | | | |
| --- | --- | --- | --- | --- |
| **JUNO residue** | **Δ Affinity, kcal/mole** | **IZUMO1 residues with VDW interactions < 4 Å** | **Additional Interactions** | **IZUMO1 Residue** |
| TYR44 | 6.4 | SER241, VAL141, TYR163, ARG160 |  |  |
| GLU45 | 4.9 | ASN239, SER241, ARG160 | H-bond, Oe2 | ARG160, Nh2 |
|  |  |  | salt bridge, Oe2 | ARG160, Nh2 |
|  |  |  | H-bond, Oe2 | ASN239, Nd2 |
| TRP62 | 20.8 | SER162, TYR163, ARG160, LYS161, VAL141 |  |  |
| LEU81 | 15.9 | TRP148, MET75, HIS157, TYR134 | H-bond, N | MET75, O |
|  |  |  | H-bond, O | TRP148, Ne1 |
| MET83 | 5.5 | TRP148, LYS153, ASN151 |  |  |
| ARG87 | 9.4 | ALA158, ARG160, HIS157 | Pi-cation | ARG160, Nh2 |
|  |  |  | H-bond, Nh1 | ALA158, O |
| TYR147 | 9.1 | LYS150 | Pi-cation | LYS150, Nz |
|  |  |  | H-bond, Oh | LYS150, O |
| LYS163 | 5.5 | GLU71 | H-bond, Nz | GLU71, Oe2 |
|  |  |  | salt bridge, Nz | GLU71, Oe2 |

| **Table S3- IVF developmental rate following small molecule treatments** | | | | | | | |  |
| --- | --- | --- | --- | --- | --- | --- | --- | --- |
| **Molecule, Concertation** | **Days Incubated** | **1 cells** | **2 cells** | **other than blastocysts** | **blastocysts** | **dead cells** | **number of oocytes** | |
| **DMSO, 100uM** | 1D |  | **100%** |  |  |  | 67 | |
|  | 4D |  |  | 29% | **46%** | 25% | 63 | |
| **Z166909640, 100uM** | 1D | 81% | **13%** |  |  | 5% | 75 | |
|  | 4D |  |  | 78% | **4%** | 18% | 73 | |
| **Z218197806, 100uM** | 1D | 67% | **22%** |  |  | 11% | 18 | |
|  | 4D |  |  | 67% | **7%** | 26% | 15 | |
| **Z826859956, 100uM** | 1D | 51% | **42%** |  |  | 7% | 55 | |
|  | 4D |  |  | 44% | **26%** | 30% | 54 | |
| **DMSO, 100uM** | 1D | 24% | **75%** |  |  | 1% | 83 | |
|  | 4D |  |  | 61% | **38%** | 1% | 84 | |
| **Z31663627, 100uM** | 1D | 36% | **63%** |  |  | 1% | 219 | |
|  | 4D |  |  | 91% | **5%** | 4% | 209 | |
| **Z73385117, 100uM** | 1D | 43% | **55%** |  |  | 2% | 150 | |
|  | 4D |  |  | 82% | **12%** | 6% | 150 | |
| **Z96915800, 100uM** | 1D | 49% | **51%** |  |  | 0% | 35 | |
|  | 4D |  |  | 83% | **17%** | 0% | 35 | |
| **DMSO, 100uM** | 1D | 20% | **80%** |  |  | 0% | 128 | |
|  | 4D |  |  | 59% | **31%** | 10% | 127 | |
| **Z218197806, 100uM** | 1D | 10% | **89%** |  |  | 1% | 144 | |
|  | 4D |  |  | 50% | **15%** | 35% | 135 | |
| **Z218197806, 10uM** | 1D | 3% | **95%** |  |  | 2% | 107 | |
|  | 4D |  |  | 31% | **40%** | 28% | 99 | |
| **Z218197806, 1uM** | 1D | 34% | **63%** |  |  | 3% | 116 | |
|  | 4D |  |  | 65% | **21%** | 14% | 112 | |
|  | | | | | | | |  |
| **Table S3- IVF developmental rate following small molecule treatments** | | | | | | | |  |
| **Molecule, Concertation** | **Days Incubated** | **1 cells** | **2 cells** | **other than blastocysts** | **blastocysts** | **dead cells** | **number of oocytes** | |
| **DMSO, 100uM** | 1D | 0% | **100%** |  |  | 0% | 38 | |
|  | 4D |  |  | 5% | **72%** | 23% | 39 | |
| **Z281538714, 100uM** | 1D | 9% | **76%** |  |  | 15% | 46 | |
|  | 4D |  |  | 5% | **65%** | 30% | 40 | |
| **Z218197806, 100uM** | 1D | 79% | **10%** |  |  | 10% | 29 | |
|  | 4D |  |  | 86% | **0%** | 14% | 29 | |
| **Z102448578, 100uM** | 1D | 29% | **63%** |  |  | 8% | 52 | |
|  | 4D |  |  | 59% | **7%** | 34% | 53 | |
| **DMSO, 100uM** | 1D | 42% | **58%** |  |  | 0% | 24 | |
|  | 4D |  |  | 46% | **46%** | 8% | 24 | |
| **Z115160130, 100uM** | 1D | 45% | **50%** |  |  | 5% | 22 | |
|  | 4D |  |  |  |  |  |  | |
| **Z1224462206, 100uM** | 1D | 49% | **43%** |  |  | 9% | 35 | |
|  | 4D |  |  | 46% | **36%** | 18% | 39 | |
| **Z95873536, 100uM** | 1D | 77% | **21%** |  |  | 3% | 34 | |
|  | 4D |  |  | 80% | **13%** | 7% | 30 | |
| **DMSO, 100uM** | 1D | 18% | **82%** |  |  | 0% | 128 | |
|  | 4D |  |  | 15% | **72%** | 13% | 131 | |
| **Z786028994, 10uM** | 1D | 59% | **38%** |  |  | 3% | 110 | |
|  | 4D |  |  | 56% | **34%** | 10% | 108 | |
| **Z786028994, 100uM** | 1D | 99% | **0%** |  |  | 1% | 99 | |
|  | 4D |  |  | 98% | **0%** | 2% | 99 | |
| **Z1033235866, 10uM** | 1D | 11% | **89%** |  |  | 0% | 37 | |
|  | 4D |  |  | 8% | **87%** | 5% | 38 | |
|  | | | | | | | |  |
| **Table S3- IVF developmental rate following small molecule treatments** | | | | | | | |  |
| **Molecule, Concertation** | **Days Incubated** | **1 cells** | **2 cells** | **other than blastocysts** | **blastocysts** | **dead cells** | **number of oocytes** | |
| **DMSO, 50uM** | 1D | 19% | **81%** |  |  | 0% | 32 | |
|  | 4D |  |  | 22% | **69%** | 9% | 32 | |
| **Z786028994, 50uM** | 1D | 85% | **11%** |  |  | 5% | 66 | |
|  | 4D |  |  | 86% | **2%** | 12% | 58 | |
| **Z66693270, 50uM** | 1D | 46% | **46%** |  |  | 7% | 41 | |
|  | 4D |  |  | 43% | **50%** | 7% | 42 | |
| **Z18823321, 50uM** | 1D | 57% | **35%** |  |  | 8% | 49 | |
|  | 4D |  |  | 62% | **19%** | 19% | 37 | |
| **DMSO, 100uM** | 1D | 58% | **40%** |  |  | 2% | 38 | |
|  | 4D |  |  | 43% | **27%** | 30% | 37 | |
| **Z49720304, 100uM** | 1D | 6% | **66%** |  |  | 29% | 35 | |
|  | 4D |  |  | 22% | **33%** | 44% | 36 | |
| **Z131775002, 100uM** | 1D | 5% | **90%** |  |  | 5% | 20 | |
|  | 4D |  |  | 15% | **60%** | 25% | 20 | |
| **Z786028994, 1uM** | 1D | 19% | **78%** |  |  | 4% | 27 | |
|  | 4D |  |  | 48% | **9%** | 43% | 23 | |
| **DMSO, 100uM** | 1D | 40% | **53%** |  |  | 7% | 53 | |
|  | 4D |  |  | 49% | **47%** | 4% | 47 | |
| **Z91134263, 100uM** | 1D | 18% | **77%** |  |  | 6% | 34 | |
|  | 4D |  |  | 65% | **23%** | 13% | 31 | |
| **Z73456907, 100uM** | 1D | 23% | **77%** |  |  | 0% | 48 | |
|  | 4D |  |  | 46% | **48%** | 7% | 46 | |
| **Z1290281203, 100uM** | 1D | 94% | **0%** |  |  | 6% | 62 | |
|  | 4D |  |  | 93% | **0%** | 7% | 61 | |
|  | | | | | | | |  |
| **Table S3- IVF developmental rate following small molecule treatments** | | | | | | | |  |
| **Molecule, Concertation** | **Days Incubated** | **1 cells** | **2 cells** | **other than blastocysts** | **blastocysts** | **dead cells** | **number of oocytes** | |
| **DMSO, 100uM** | 1D | 55% | **45%** |  |  | 0% | 11 | |
|  | 4D |  |  | 38% | **25%** | 37% | 8 | |
| **Z109481864, 100uM** | 1D | 58% | **36%** |  |  | 6% | 53 | |
|  | 4D |  |  | 58% | **36%** | 6% | 53 | |
| **Z73456907, 100uM** | 1D | 0% | **100%** |  |  | 0% | 24 | |
|  | 4D |  |  | 9% | **74%** | 17% | 23 | |
| **Z1290281203, 100uM** | 1D | 100% | **0%** |  |  | 0% | 30 | |
|  | 4D |  |  | 100% | **0%** | 0% | 20 | |
| **DMSO, 1uM** | 1D | 9% | **82%** |  |  | 9% | 75 | |
|  | 4D |  |  | 19% | **52%** | 29% | 72 | |
| **Z1290281203, 0.01uM** | 1D | 15% | **73%** |  |  | 12% | 75 | |
|  | 4D |  |  | 16% | **55%** | 29% | 69 | |
| **Z1290281203, 0.1uM** | 1D | 5% | **93%** |  |  | 2% | 56 | |
|  | 4D |  |  | 21% | **55%** | 24% | 53 | |
| **Z1290281203, 1uM** | 1D | 31% | **54%** |  |  | 15% | 78 | |
|  | 4D |  |  | 38% | **37%** | 25% | 71 | |
| **DMSO, 100uM** | 1D | 50% | **42%** |  |  | 8% | 62 | |
|  | 4D |  |  | 35% | **56%** | 9% | 80 | |
| **Z1172207733, 100uM** | 1D | 78% | **22%** |  |  | 0% | 36 | |
|  | 4D |  |  | 78% | **19%** | 3% | 36 | |
| **Z49734016, 100uM** | 1D | 39% | **46%** |  |  | 15% | 65 | |
|  | 4D |  |  | 37% | **48%** | 15% | 62 | |
| **Z56788505, 100uM** | 1D | 69% | **19%** |  |  | 12% | 68 | |
|  | 4D |  |  | 72% | **11%** | 17% | 65 | |
|  | | | | | | | |  |
| **Table S3- IVF developmental rate following small molecule treatments** | | | | | | | |  |
| **Molecule, Concertation** | **Days Incubated** | **1 cells** | **2 cells** | **other than blastocysts** | **blastocysts** | **dead cells** | **number of oocytes** | |
| **DMSO, 100uM** | 1D | 60% | **28%** |  |  | 12% | 50 | |
|  | 4D |  |  | 66% | **14%** | 20% | 50 | |
| **Z1021013936, 100uM** | 1D | 44% | **48%** |  |  | 9% | 23 | |
|  | 4D |  |  | 70% | **17%** | 13% | 23 | |
| **Z738918766, 100uM** | 1D | 56% | **27%** |  |  | 17% | 59 | |
|  | 4D |  |  | 63% | **10%** | 27% | 59 | |
| **DMSO, 100uM** | 1D | 26% | **72%** |  |  | 2% | 43 | |
|  | 4D |  |  | 33% | **38%** | 29% | 42 | |
| **Z102415690, 100uM** | 1D | 38% | **49%** |  |  | 13% | 61 | |
|  | 4D |  |  | 56% | **27%** | 17% | 59 | |
| **Z225172500, 100uM** | 1D | 12% | **77%** |  |  | 11% | 65 | |
|  | 4D |  |  | 76% | **11%** | 13% | 63 | |
| **Z194601086, 100uM** | 1D | 24% | **72%** |  |  | 4% | 29 | |
|  | 4D |  |  |  |  |  |  | |
| **DMSO, 100uM** | 1D | 49% | **36%** |  |  | 15% | 70 | |
|  | 4D |  |  | 61% | **7%** | 32% | 69 | |
| **Z17802263, 100uM** | 1D | 70% | **8%** |  |  | 22% | 50 | |
|  | 4D |  |  | 72% | **6%** | 22% | 50 | |
| **Z234901630, 100uM** | 1D | 71% | **20%** |  |  | 8% | 59 | |
|  | 4D |  |  | 77% | **11%** | 12% | 57 | |
| **Z751761886, 100uM** | 1D | 84% | **4%** |  |  | 12% | 43 | |
|  | 4D |  |  | 83% | **0%** | 17% | 41 | |

| **Table S4: Composition of high calcium HTF medium** | | | |
| --- | --- | --- | --- |
| **Reagent Name** | **mg/100ml** | **Vendor** | **Cat. number** |
| **NaCl** | 593.8 | Sigma | S-5886 |
| **KCl** | 35.0 | Sigma | P-5405 |
| **MgSO4·7H2O** | 4.9 | Sigma | M-7774 |
| **KH2PO4** | 5.4 | Sigma | P-5655 |
| **CaCl2·2H2O** | 75.5 | Sigma | C-7902 |
| **NaHCO3** | 210.0 | Sigma | S-5761 |
| **Glucose** | 50.0 | Sigma | G-6152 |
| **Na-lactate (ml)*** | 0.34 | Sigma | L-7900 |
| **Na-Pyruvate** | 3.7 | Sigma | P-4562 |
| **Penicillin G** | 7.5 | Sigma | P-4687 |
| **Streptomycin** | 5.0 | Sigma | S-1277 |
| **BSA (Fraction V, Fatty Acid-Free)** | 400.0 | Merck | 126575 |
| **Phenol Red (0.5% soln)** | 0.04(ml) | Sigma | P-0290 |

| **Table S5: Composition of high calcium TYH+MBCD sperm pre-incubation medium** | | | |
| --- | --- | --- | --- |
| **Reagent Name** | **mg/100ml** | **Vendor** | **Cat. number** |
| **NaCl** | 697.6 | Sigma | S-5886 |
| **KCl** | 35.6 | Sigma | P-5405 |
| **MgSO4·7H2O** | 29.3 | Sigma | M-7774 |
| **KH2PO4** | 16.2 | Sigma | P-5655 |
| **NaHCO3** | 210.6 | Sigma | S-5761 |
| **Na-Pyruvate** | 5.5 | Sigma | P-4562 |
| **Glucose** | 100.0 | Sigma | G-6152 |
| **CaCl2·2H2O** | 25.1 | Sigma | C-7902 |
| **Methyl-β-cyclodextrin** | 98.3 | Sigma | C-4555 |
| **Penicillin G** | 7.5 | Sigma | P-4687 |
| **Streptomycin** | 5.0 | Sigma | S-1277 |
| **Polyvinylalcohol** | 100.0 | Sigma | P-8136 |
|  |  |  |  |

**
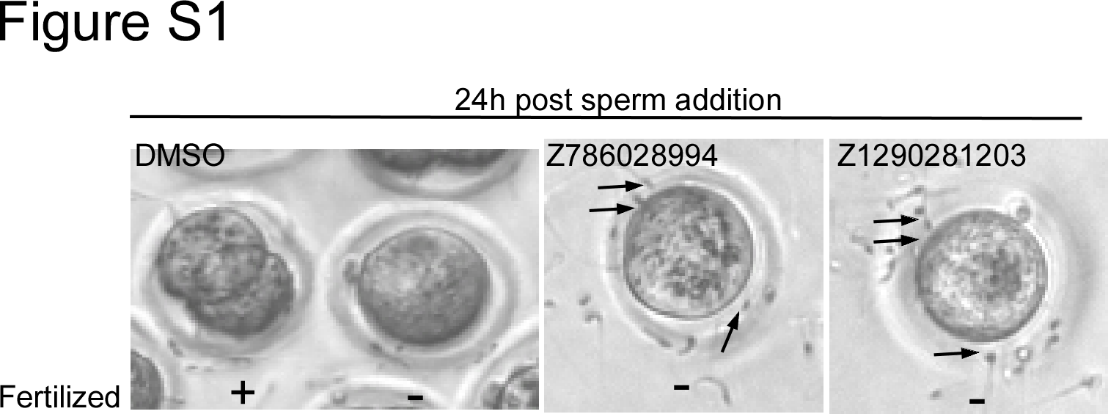
**

**Figure S1. Sperm accumulate at the perivitelline space of Z786028994 and Z1290281203 pre-treated**

**oocytes.**

Phase contrast images of oocytes one-day post fertilization. While in the DMSO pre-treated control oocytes

no sperm is found inside the perivitelline space of both fertilized and non-fertilized eggs (left panel), in the

pre-treated Z786028994 and Z1290281203 oocytes, multiple sperm cells can be seen in the perivitelline

space (middle panel and right panel, respectively). Arrow mark sperm penetration into the perivitelline

space.
